# Alginate oligosaccharides can maintain activities of lysosomes under low pH condition

**DOI:** 10.1038/s41598-021-91175-6

**Published:** 2021-06-01

**Authors:** Ra-Mi Park, Ngoc-Han Thi Nguyen, Su-Min Lee, Yang-Hoon Kim, Jiho Min

**Affiliations:** 1 Graduate School of Semiconductors and Chemical Engineering, Jeonbuk National University, 567 Baekje-daero, Deokjin-gu, Jeonju-si, Jeollabuk-do 54896 Republic of Korea; 2 Department of Bioprocess Engineering, Jeonbuk National University, 567 Baekje-daero, Deokjin-gu, Jeonju-si, Jeollabuk-do 54896 Republic of Korea; 3 School of Biological Sciences, Chungbuk National University, Chungdae-Ro, Seowon-Gu, Cheongju, 28644 South Korea

**Keywords:** Biological techniques, Biotechnology

## Abstract

The objective of this study was to report that lysosome extracted from egg white could be used as a drug through oral administration for treating diseases by using pH sensitive alginate oligosaccharides. Lysosome-alginate oligosaccharides composite were formulated for oral administration of lysosomes. The dissolution test confirmed the availability of the oral dosage form. When lysosome were used as an independent drug, the activity of protein was lost due to influence of low pH. Its antibacterial activity was also remarkably reduced. However, when lysosome-alginate oligosaccharides composite form was used, antimicrobial activity of lysozyme was maintained. At low pH, a gel-like matrix was formed by alginate oligosaccharides to protect the lysosome. When the pH was increased, alginate oligosaccharides were dissolved and the lysosome was released. SDS–polyacrylamide gel electrophoresis analysis of released lysosomes revealed that alginate oligosaccharide could effectively protect the lysosome from degradation or hydrolysis under acidic conditions for at least 2 h. The results of this study are important for application of lysosomes as therapeutic agents, and also it was confirmed that alginate oligosaccharides have potential as direct delivery system for the oral application of protein derived therapies.

## Introduction

Lysosomes are cell organelles containing various hydrolytic enzymes capable of digesting intracellular and extracellular materials. Lysosomes are abundant in egg whites. Egg white contains many biologically active proteins (54% albumine, 13% ovotransferin, 11% ovomucoid, and 3.4% lysozyme)^1^ that are antimicrobial, antiviral, antiphlogistic, and antalgic^2^. In particular, lysozymes can function as hydrolytic enzymes that can degrade bacteria cell walls^3,4^. In drug delivery studies, oral route of administration has high acceptability and patient compliance with the advantage of self-administration^5^. Although steady development of drug delivery technology is underway, much research on the oral administration of protein drugs is still needed. After oral administration, protein drugs face several problems, including rapid systemic degeneration/degradation and malabsorption in the small intestine^6^. In order to orally administer a protein drug, it is necessary to provide a drug that improves the absorption of the drug by increasing the reversible permeability of the mucosal epithelium and maintains efficacy without protein denaturation^7^. Therefore, delivery systems are needed to increase the bioavailability of these drugs, and gastrointestinal transit time is one of the challenges to overcome, especially in the development of sustained-release formulations^8^. This has led to considerable research focusing on overcoming drug's retention time problem at low pH for effective oral delivery of the drug^5^.

Alginates (alginic acid, sodium alginate, and potassium alginate) have been extensively explored as mucoadhesive biomaterials owing to their very good cytocompatibility, biocompatibility, biodegradation^9^, sol–gel transition properties, and chemical versatility that make it possible to further modify them to tailor their properties^10^. Alginate gels tend to be eroded under more neutral and basic pH values than under acid conditions^11^. This property has motivated its use in chemical stabilization of drugs and biologicals of oral administration that are unstable in gastric fluids.

In general, AOs has a short chain length, so it has improved water solubility when compared to high molecular weight alginates of the same monomer^12^. Since alginate oligosaccharides (AOs) are considered as non-toxic, non-immunogenic and biodegradable polymers, they are attractive candidates in biomedical applications^13^. AOs are linear polymers of polysaccharides with alternating gel formation properties consisting of β-(1-4)-D-mannosyluronic acid and α-(1-4)-L-glucosyluronic acid. AOs exhibit important biological activities not only in mammals, but also in plants. AOs have additional properties such as antimicrobial effects^14,15^, anti-inflammatory effects^16^, defense-enhancing effects against infection by certain pathogens^17^, and anti-glycating effects^18^. AOs can promote growth and proliferation of human cells, including keratinocytes^19–21^ and endothelial cells^22^. In addition, they can potentially protect nerve cells from oxidative stress^23^. Finally, AOs have interesting properties for fighting microbial pathogens. AOs can potentiate the action of selected antibiotics by disturbing multidrug-resistant bacteria^24^. Similarly, they can inhibit fungal cell growth and enhance activities of antifungal agents against *Candida* and *Aspergillus* species^25^. Here, the purpose of this study was to confirm that the lysosome isolated from egg white can be used as an eco-friendly agent for the treatment of metabolic diseases related to microorganisms living in the intestine through oral drug delivery using pH-sensitive AO.

## Results

### Formation of lysosome-AOs composite

**Figure 1:**
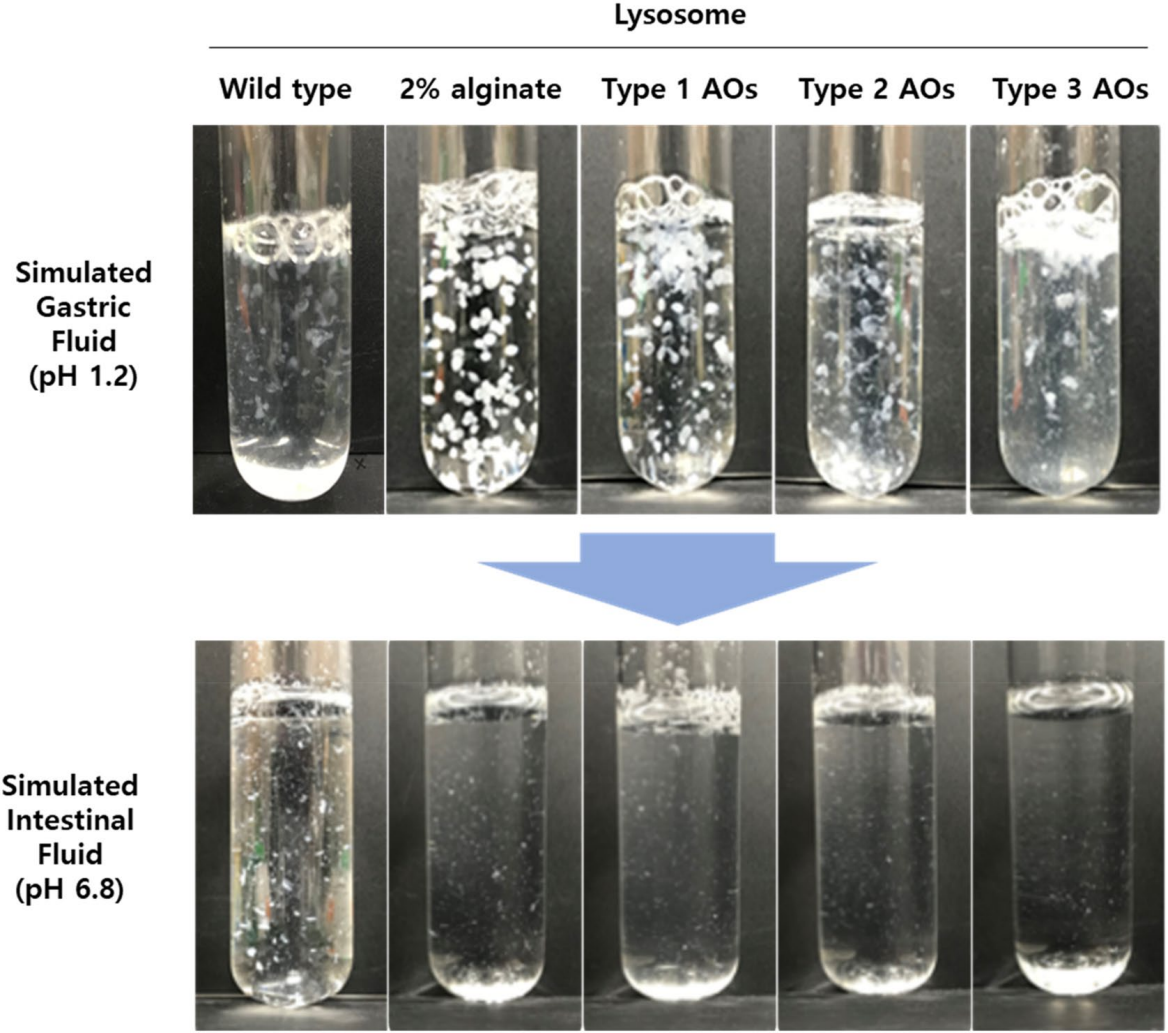
Appearance of lysosomal suspension immobilized with a pH-sensitive biopolymer in simulated gastric and intestine environment. Type 1 AOs, Type 2 AOs, and Type 3 AOs refer to alginate oligosaccharides made by lyase treatment for 6 h, 12 h, and 24 h, respectively.

Three types of alginate oligosaccharides used in this study were depolymerized with alginate by treating sodium alginate at 37 °C for 6 h, 12 h, and 24 h with alginate decomposing enzyme, respectively, to prepare three types of alginate oligosaccharides. The prepared alginate oligosaccharide was analyzed by MALDI-TOF–MS spectroscopy, and each alginate oligosaccharide had an intensity of 379.25 m/z peak corresponding to [M + Na] as 41.96%, 72.03%, and 88.86%. Results of observing the appearance of wild type lysosome, lysosome-2% alginate composite, or lysosome-AOs composite under low pH environment are shown in Fig. 1. Wild type lysosomes without alginate or AOs were used as negative controls. Wild type lysosomes were almost the same before and after the dissolution test. Denaturation of protein was observed in simulated gastric fluid due to its low pH. Lysosome-2% alginate composite was used as a positive control. In acid phase (simulated gastric juice, pH 1.2), alginate gel containing lysosome was formed because pH-sensitive alginate could form spherical lysosome-2% alginate. In the buffer phase (simulated intestinal juice, pH 6.8), the alginate gel dissolved to release lysosome. Next, lysosome-AOs (lysosome-Type 1 AOs, lysosome-Type 2 AOs, lysosome-Type 3 AOs) composites formed spherical gel containing lysosome at low pH. In the buffer phase, the gel formed by AOs was dissolved and lysosome was released. Among these three types of AOs, Type 1 AOs behaved like alginate the most.

### Antimicrobial activity of the released lysosome

**Figure 2:**
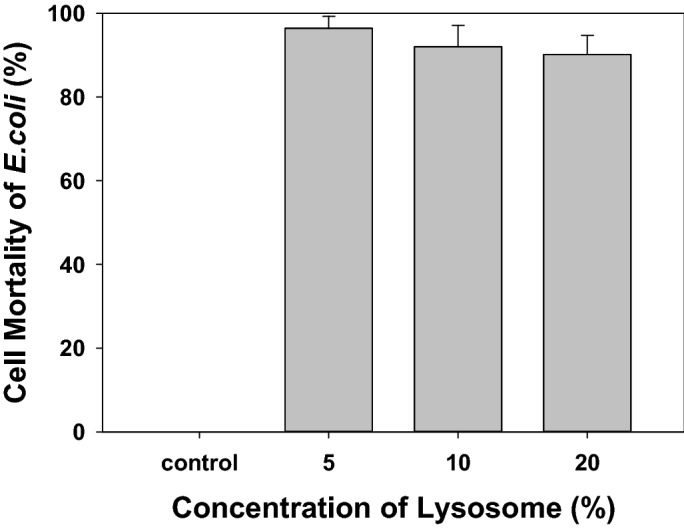
Antimicrobial activity of isolated lysosome from egg wihte.

**Figure 3:**
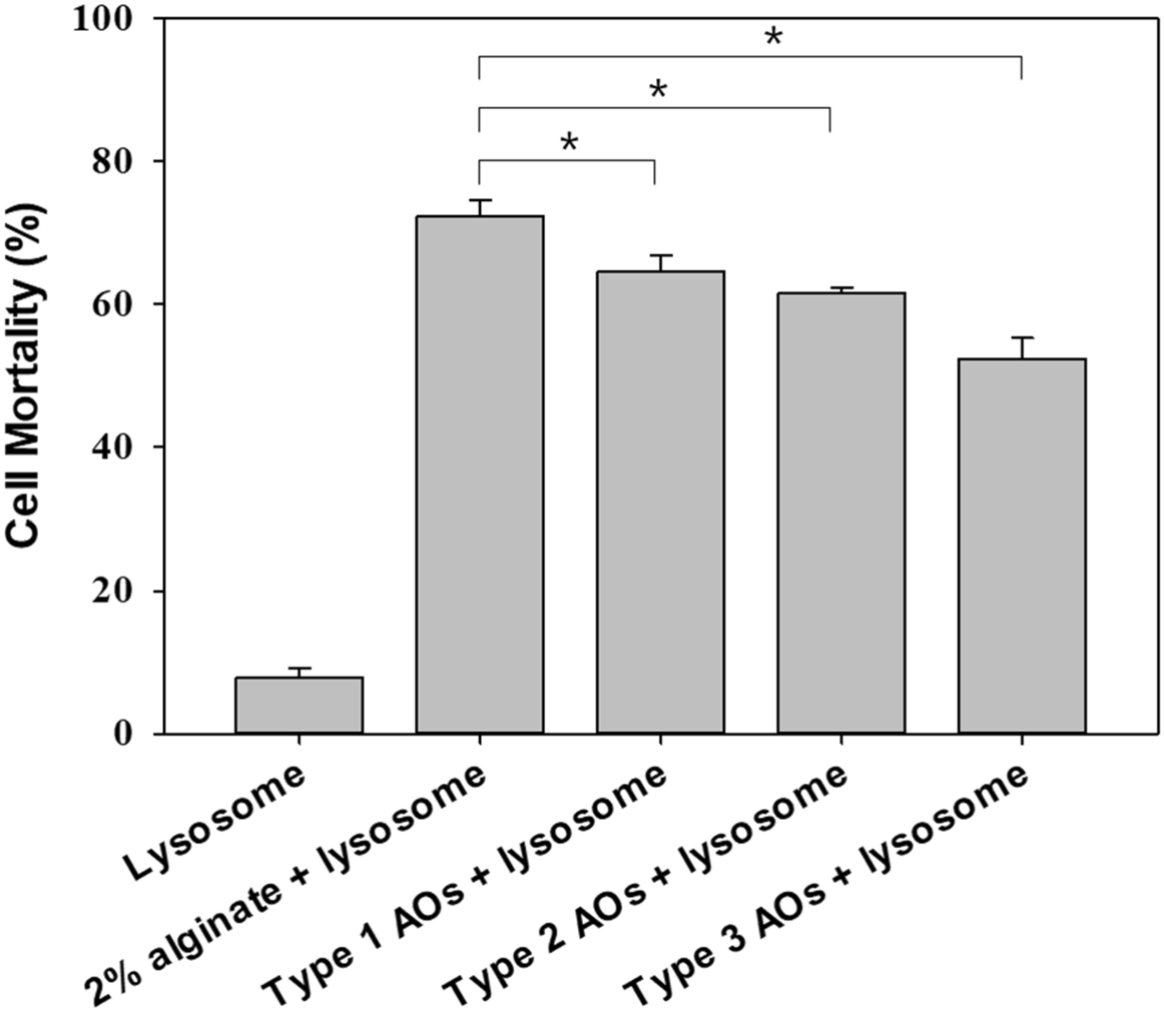
Antimicrobial activity of lysosomes immobilized on pH-sensitive biopolymers passing simulated gastrointestinal environment against *Escherichia coli*. In the graph, lysosomes without any treatment, lysosomes immobilized on 2% alginate, and antimicrobial activities against three types of AO-immobilized lysosomes are shown in order.

To use lysosomes as a drug, lysosome-2% alginate composite and lysosome-AOs composite were manufactured and applied as oral delivery systems. The antimicrobial activity of wild type lysosome against cell mortality of *E. coli* was 95% or more (Fig. 2). Figure 3 shows results of dissolution test, confirming the antimicrobial activity of lysosomes released into the intestine. Wild type lysosomes used for the dissolution test showed approximately 80% decreased antimicrobial activity. However, the antimicrobial activity of the lysosomes used in the dissolution test was maintained after mixing 2% alginate with three types of AOs. The antimicrobial activity of lysosome mixed with 2% alginate was the highest, followed by lysosome mixed with Type 1 AOs.

### Protein of the released lysosome

**Figure 4:**
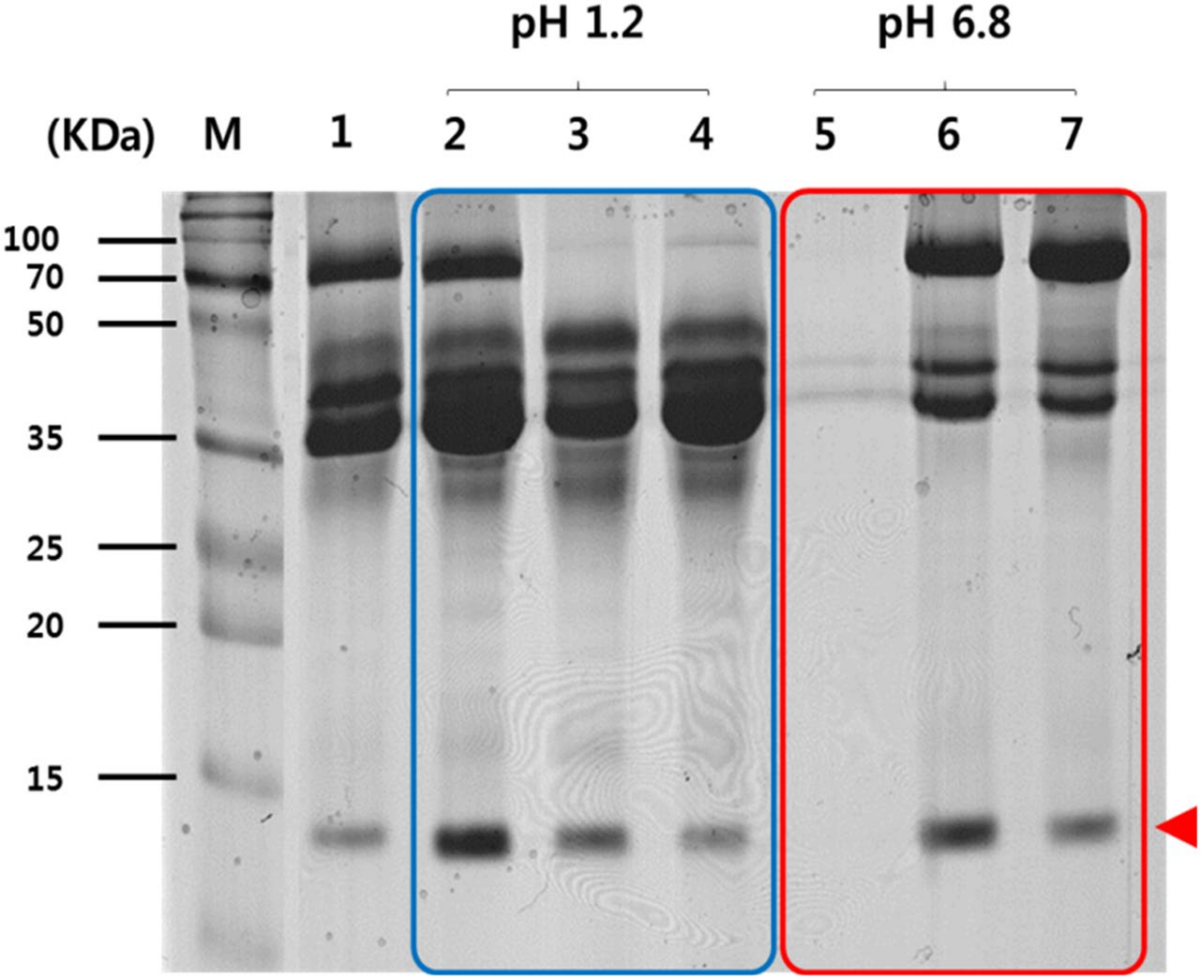
Expression patterns of lysosomal protein in egg white lysosome. Arrow indicates lysozyme. Lane 1 indicates total protein of wild type. Lanes 2, 3, and 4 indicate total proteins isolated from wild type, lysosome-2% alginate composites, and lysosome-AOs composites from Simulated Gastric Fluid. Lanes 5, 6, and 7 indicate total proteins isolated from wild type, lysosome-2% alginate composites, and lysosome-AOs composites from Simulated Intestinal Fluid.

Figure 4 shows SDS-PAGE results. Lane M shows protein marker (DokDo-MARK). Lane 1 shows lysosomal protein extracted from wild type lysosome (without alginate or AOs) as a control. Lanes 2 to 4 schematically describe lysosomal protein patterns extracted from wild type lysosome, lysosome-2% alginate composite, and lysosome-AOs composites formed in simulated gastric fluid (pH 1.2). As a result, the pattern was similar to that shown in lane 1. Lanes 1 and 2 and lanes 3 and 4 showed almost the same pattern. After reaction in simulated gastric fluid, wild type lysosome, lysosome-2% alginate composites, and lysosome-AOs composites were collected and reacted in simulated intestinal fluid. Lanes 5, 6, and 7 show lysosomal protein patterns extracted from wild type lysosomes, lysosome-2% alginate composites, and lysosome-AOs composites dissolved in simulated intestinal fluid (pH 6.8), respectively. As a result, protein pattern extracted from the wild type lysosome (without alginate/AOs) appeared in simulated gastric fluid. This protein pattern did not appear in simulated intestinal fluid. On the other hand, lanes 6 and 7 were lysosomal proteins extracted from lysosome-2% alginate composites and lysosome-AOs composites dissolved and released in simulated intestinal fluid, respectively. Although expression levels and patterns were slightly changed, these results were similar to those under acidic conditions. In particular, lysozyme is another significant protein discovered in egg white. The molecular weight of lysozyme is 14,400 Da. Lysozyme consists of a single polypeptide chain with 129 amino acids^25^. These results confirmed that the pattern of protein extracted from released lysosome appeared below 15 kDa molecular weight (Fig. 4, arrow). In other words, sodium dodecyl sulfate–polyacrylamide gel electrophoresis (SDS-PAGE) analysis showed that mixed alginate and alginate oligosaccharides could effectively protect lysosomes from degradation or hydrolysis under acidic conditions for at least 2 h.

### Morphology of complex

**Figure 5:**
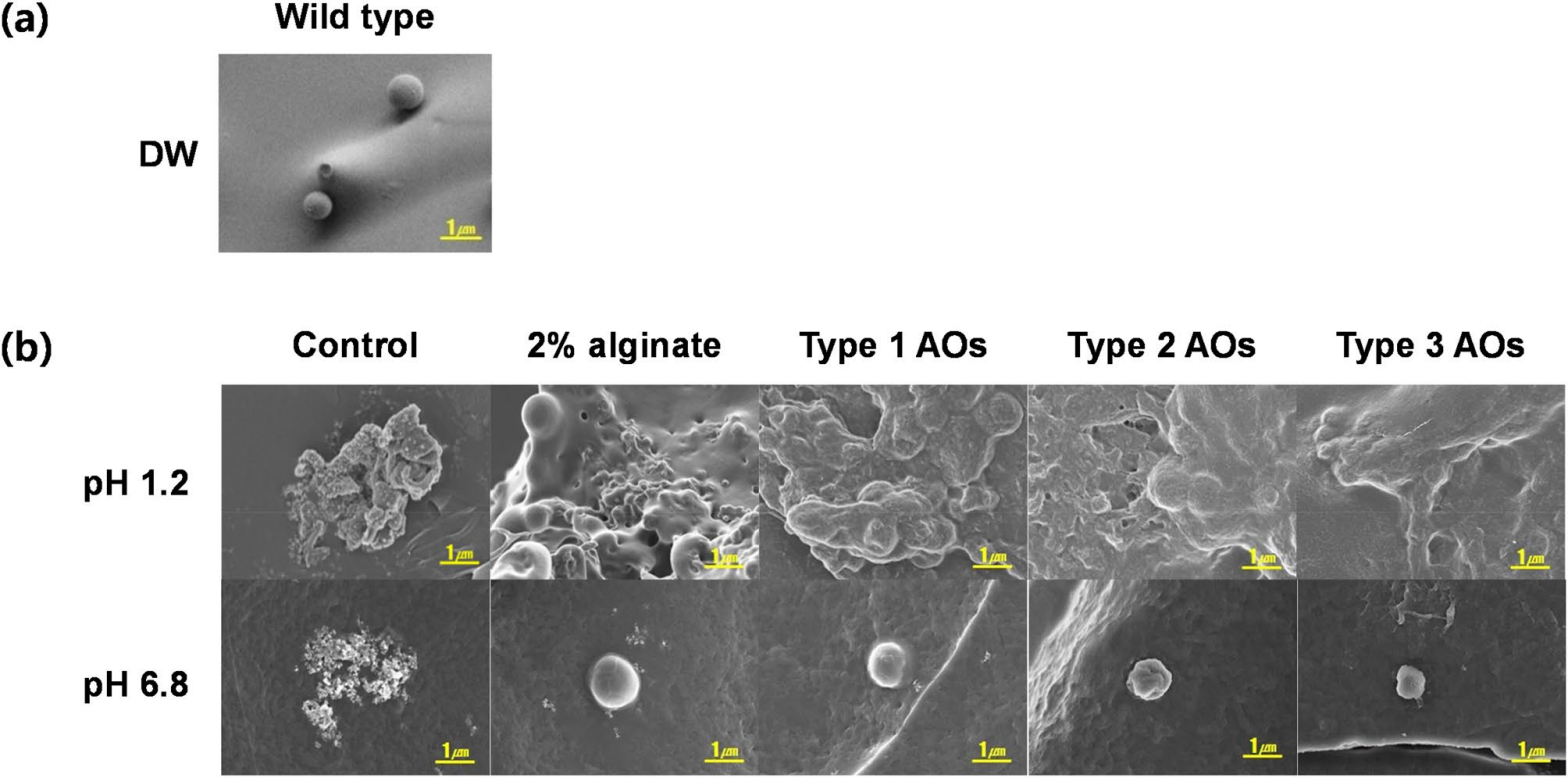
Field emission scanning electron microscopy (FE-SEM) images of morphology. (**a**) Wild type lysosome (unused for experiments) was observed in DW. (**b**) Morphology formed by oral administration of lysosomes. Scale bar is 1 μm.

Figure 5 shows morphologies of lysosomes observed according to environmental conditions during oral administration. In Fig. 5a, wild type lysosome (unused for experiments) in DW (distilled water) was observed. Figure 5b confirmed the morphology formed by oral administration of lysosomes. The picture showed lysosomes in pH 1.2 solution (instead of gastric fluid, and lysosomes in pH 6.8 solution (instead of intestinal fluid). As shown in Fig. 5b, the control group (without alginate or AOs) did not have spherical lysosomes at pH 1.2 or pH 6.8. On the other hand, in the case of treatment of 2% alginate with Type 1 AOs, Type 2 AOs, or Type 3 AOs, it was found that spheres seen as lysosomes in pH 1.2 were more clustered by surrounding substrates. Further, at pH 6.8, a spherical lysosome was found independently.

## Discussion

Alginate hydrogel exists in the units of D-mannuronic acid and L-guluronic acid, and is a pH-sensitive biopolymer. Alginate has a sol–gel transition characteristic, and Alginate gels tend to be eroded under more neutral and basic pH values than under acid conditions^27^. In other words, alginate hydrogel is an ideal matrix for use in oral sustained release drugs^28^. To overcome the drawback of using the properties of alginate hydrogels but not being degraded in vivo, this study proposes alginate oligosaccharides as a drug matrix in oral sustained release formulations^9^. In this study, we devised a method to utilize low molecular weight AOs to use lysosomes as oral drugs. Lysosome-AOs (lysosome-Type 1 AOs, lysosome-Type 2 AOs, lysosome-Type 3 AOs) composites formed spherical gel containing lysosome at low pH. In the buffer phase, the gel formed by AOs was dissolved and lysosome was released. Of these three types of AO, type 1 AO behaves like the alginate, making gel formation most effective. Alginate has the property of changing to alginate sol form under acidic conditions. Similarly, it was confirmed that AOs changed to sol form under acidic conditions, so that AOs act as a substrate surrounding lysosomes.

Dissolution testing is the most common method for evaluating oral modified release delivery systems for colon-specific drug delivery. In-vitro evaluation of oral modified drug delivery systems is generally performed by using standard process procedures or by dissolution testing as appropriate^29^. The dissolution test confirmed that the lysosome activity was maintained up to the intestine when the lysosomes were used for oral administration in the form of AOs complex.

Lysosome activity mediates several processes for cell feeding and antimicrobial defense, including lysosomal fusion with endosomes and phagosomes^2,30^. Thus, lysosomal enzymes are capable of cell digestion^31^. Antimicrobial effects of lysosomes isolated from egg white on different strains including Gram-positive and Gram-negative bacteria have been reported^32^. Wild type lysosomes used for the dissolution test showed remarkably decreased antimicrobial activity than before it was used in the experiment. However, the antimicrobial activity of the lysosomes used in the dissolution test was maintained after mixing 2% alginate or three types of AOs. The antimicrobial activity of lysosome mixed with 2% alginate was the highest, followed by lysosome mixed with Type 1 AOs. Therefore, it can be seen that it is effective to use type 1 AOs together to maintain lysosomal activity up to the intestine.

Lysozyme is another significant protein discovered in egg white. The molecular weight of lysozyme is 14,400 Da. Lysozyme consists of a single polypeptide chain with 129 amino acids^26^. In this study, it was shown that lysosomes mixed with alginate or alginate oligosaccharide were protected so that antibacterial activity could be maintained in acidic conditions (artificial gastric juice) for at least 2 h.

## Conclusions

The purpose of this research was to describe that lysosome isolated from egg white could be used as an environmentally friendly antimicrobial agent for treating diseases through oral drug delivery. This study applied sol–gel transition characteristics of biopolymer by pH change. AOs, when processed together with lysosome to use lysosome as a drug, allows drug release in the intestine with a high pH and limits drug exposure in the upper part of the gastrointestinal tract at low pH. Although results described in this study are from preliminary experiments for applying lysosomes as therapeutic agents, AOs have possibility as delivery systems for direct application of drugs, proteins, and lysosomes.

## Materials and methods

### Isolation of lysosome from egg white

Lysosome isolation was performed by homogenizing egg white followed by centrifugation twice under different conditions. First, egg white was homogenized for 30 min, divided into centrifuge bottles, and centrifuged at 1000 × *g* for 10 min at 4 °C. The supernatant was collected, homogenized for 2 h, and centrifuged at 20,000 × *g* for 30 min at 4 °C. After the second centrifugation, the supernatant was discarded and only the remaining pellet was collected. The collected pellet was tested for antimicrobial activity and used as lysosomes in all subsequent experiments. The collected pellet was stored at 4 °C in the dark.

### AOs production by enzymatic degradation of sodium alginate

To prepare Type 1 AOs, Type 2 AOs, and Type 3 AOs, alginate lyase (Sigma-Aldrich, A1603) was used to catalyze the cleavage of alginate. To make Type 1 AOs, 2% (w/v) of sodium alginate (Sigma-Aldrich, A0682) was prepared by dissolving sodium alginate in 100 ml of distilled water (DW) followed by incubation at 37 °C for 6 h in the presence of 20 U/mg alginate lyase in a rotary shaker at 150 rpm. After the incubation, alginate lyase in the sample was inactivated at 100 °C for 10 min and removed after centrifugation at 12,000×*g* for 10 min at 4 °C^33^. Type 2 and Type 3 AOs were prepared with the same method except that alginate lyase treatment time was changed from 6 to 12 h for Type 2 AOs and 24 h for Type 3 AOs. Fractions of AOs were collected, freeze-dried, and store at − 4 °C.

### Formation of lysosome-AOs composite using three types of AOs

Lysosomes and 2% alginate, or lysosomes and AOs were mixed at a ratio of 1:1 and prepared separately. Wild type lysosome was mixed with DW (ratio 1:1). A spherical composite was formed by dropping wild type lysosome, lysosome-2% alginate, or lysosome-AOs into a 1 ml disposable plastic syringe (Ormond Beach, FL, USA) in a simulated gastric fluid (pH 1.2). Particles formed in the acid solution were separated by centrifugation at 3000 rpm for 5 min.

### Dissolution test of lysosome-AOs composite

Dissolution test was carried out under two conditions, acid stage (simulated gastric fluid; pH 1.2) and buffer stage (simulated intestinal fluid; pH 6.8), to confirm the role of gastric-resistant coating agent or the effect of coating on drug diffusion. A dissolution study of lysosome-AOs composite was performed according to the Korean Food and Drug Administration (KFDA). To make simulated gastric fluid, 0.4 g of NaCl was dissolved in 200 ml of DW and the pH was adjusted to 1.2 using HCl. Then 25 ml of 0.2 M KH_2_PO_4_ and 11.8 ml of 0.2 N NaOH were mixed with DW to prepare 100 ml of simulated intestinal fluid. Dissolution test was performed with simulated gastric fluid of pH 1.2 at 50 rpm for 2 h at 37 °C. After 2 h, simulated gastric fluid was discarded and simulated colon fluid of pH 6.8 was added. The dissolution test was continued under the same conditions as acidic conditions for 1 h. After the dissolution test, the release of lysosome was confirmed by antimicrobial test and protein pattern analysis of lysosomal protein.

### Confirmation of antimicrobial activity of the released lysosome

To confirm the antimicrobial activity of the lysosome released to the colon, lysosomes isolated from wild type lysosome composite, lysosome-2% alginate composite, and three types of lysosome-AOs composite were used. *Escherichia coli* BL21 was used for antimicrobial activity tests. For antimicrobial test, after *E. coli* grew up to OD_600_ at 0.7–0.8, cultures were diluted to 10^6^ cells/mL with DW. After that, 100 µl of diluted *E. coli* was mixed with 900 µl lysosome and 100 µl of the mixture was spread onto LB agar plate. The antimicrobial activity of lysosome was measured using colony counts. Results are expressed as the percentage cell mortality (%) according to reference^34^.

### Analysis of proteins extracted from released lysosome

To analyze lysosomal proteins during drug delivery, proteins were extracted from wild type lysosomes, lysosome-2% alginate composite, and lysosome-AOs composite in simulated gastric fluid and intestinal fluid condition followed by SDS-PAGE. Lysosomal proteins were extracted from separated lysosomes. In this experiment, lysis buffer (0.1% NP-40, 0.5 mM DTT, 0.1 mM PMSF) was used. After wild type lysosome and lysis buffer were mixed at a ratio of 1:1, vortexing and cooling were repeated for 20 min and stabilized on ice for 30 min. Finally, we performed centrifugation at 13,000 rpm, 10 min, and 4 °C and collected separated supernatants containing lysosomal proteins extracted from egg white. After filtration of protein through 10 K Amicon Ultra filter, protein concentration was measured with Bradford assay using BSA (bovine serum albumin) as control. Proteins in the gastrointestinal tract were prepared in the same way.

To analyze protein pattern, the amount of lysosomal protein extracted from wild type lysosome, lysosome-2% alginate composite, or lysosome-AOs composite was adjusted 20 µg. Protein was denatured with 5 × SDS-PAGE sample buffer and boiled at 100 °C for 5 min. Then 12.5% separating gel was used to separate proteins by electrophoresis for 3 h. The gel was stained with Coomassie Brilliant Blue overnight and de-stained with de-staining buffer (45% methanol, 10% acetic acid) until Coomassie Brilliant Blue stain was removal.

### Observation of the surface by Field emission scanning electron microscope

Surfaces of wild type lysosome, lysosome-2% alginate composite, and lysosome-Types 1, 2, and 3 AOs composite under different pH conditions were observed by Field emission scanning electron microscope (FE-SEM) (Carl Zeiss SUPRA, Germany). Samples were observed after freeze-drying and storage at − 4 °C.

### Data analysis

Each data point was taken from three independent samples that were run simultaneously for error analysis. Means were reported to correlate with the standard deviation of some experimental conditions. The data was analyzed using SigmaPlot (Systat Software, Inc., USA). A p-value < 0.05 was considered significant.

## Data Availability

All the data generated and analyzed during this study are included in the published article.

## Abbreviations

AOs: Alginate Oligosaccharides
SDS-PAGE: Sodium dodecyl sulfate—polyacrylamide gel electrophoresis
DW: Distilled water
